# Structural Hippocampal Anomalies in a Schizophrenia Population Correlate with Navigation Performance on a Wayfinding Task

**DOI:** 10.3389/fnbeh.2014.00088

**Published:** 2014-03-14

**Authors:** Andrée-Anne Ledoux, Patrice Boyer, Jennifer L. Phillips, Alain Labelle, Andra Smith, Véronique D. Bohbot

**Affiliations:** ^1^University of Ottawa Institute of Mental Health Research, Ottawa, ON, Canada; ^2^School of Psychology, University of Ottawa, Ottawa, ON, Canada; ^3^Université Paris Diderot – Paris 7, Paris, France; ^4^Douglas Mental Health University Institute, McGill University, Montreal, QC, Canada

**Keywords:** psychiatric population, VBM, allocentric strategy, episodic memory, spatial memory, hippocampus

## Abstract

Episodic memory, related to the hippocampus, has been found to be impaired in schizophrenia. Further, hippocampal anomalies have also been observed in schizophrenia. This study investigated whether average hippocampal gray matter (GM) would differentiate performance on a hippocampus-dependent memory task in patients with schizophrenia and healthy controls. Twenty-one patients with schizophrenia and 22 control participants were scanned with an MRI while being tested on a wayfinding task in a virtual town (e.g., find the grocery store from the school). Regressions were performed for both groups individually and together using GM and performance on the wayfinding task. Results indicate that controls successfully completed the task more often than patients, took less time, and made fewer errors. Additionally, controls had significantly more hippocampal GM than patients. Poor performance was associated with a GM decrease in the right hippocampus for both groups. Within group regressions found an association between right hippocampi GM and performance in controls and an association between the left hippocampi GM and performance in patients. A second analysis revealed that different anatomical GM regions, known to be associated with the hippocampus, such as the parahippocampal cortex, amygdala, medial, and orbital prefrontal cortices, covaried with the hippocampus in the control group. Interestingly, the cuneus and cingulate gyrus also covaried with the hippocampus in the patient group but the orbital frontal cortex did not, supporting the hypothesis of impaired connectivity between the hippocampus and the frontal cortex in schizophrenia. These results present important implications for creating intervention programs aimed at measuring functional and structural changes in the hippocampus in schizophrenia.

## Introduction

Cognitive dysfunction is believed to be among the core features of schizophrenia. Despite abundant evidence of a prefrontal impairment in schizophrenia, this type of deficit is not specific to schizophrenia and has been extensively reported in different psychiatric disorders (e.g., mood disorders, OCD spectrum; Clark et al., 2010). Emerging research indicates that other types of deficits are more characteristic of schizophrenia, such as an episodic memory deficit, thought to be related to a dysfunction at the level of the hippocampal formation (Aleman et al., 1999; Boyer et al., 2007).

One of the most robust findings in schizophrenia is the abnormal hippocampal structure (Weiss et al., 2005). Abundant evidence from post-mortem evaluations (Bogerts et al., 1990) and *in vivo* magnetic resonance imaging (MRI) studies has demonstrated a reduced volume of the hippocampal regions (Nelson et al., 1998; Wright et al., 2000). These MRI findings have also been observed in prodromal and first episode subjects (Pantelis et al., 2003). Structural MRI studies have demonstrated that the hippocampal volume deficit is diffused throughout the anterior and posterior portions and not localized to any given parts of the hippocampus (Weiss et al., 2005). Studies have demonstrated shape differences (Shenton et al., 2002) in the hippocampal structure but also morphological differences (size, organization, and shape) in the hippocampal neurons. Post-mortem studies have demonstrated reduced neuronal size (Benes et al., 1991; Arnold et al., 1995; Zaidel et al., 1997) and disorganized pyramidal cell (Luts et al., 1998) in the hippocampus proper subfields CA1 (Arnold et al., 1995), CA2, and CA3 (Zaidel et al., 1997) and subiculum (Kovelman and Scheibel, 1984; Arnold et al., 1995). Further, there have been reports of decreased density of dendritic spines, and less extensive apical dendritic trees in the pyramidal neurons of the subicular complex (Rosoklija et al., 2000) and in the granule cells of the dentate gyrus (Lauer et al., 2003).

It is now commonly accepted that the hippocampus plays a critical role in learning, long-term memory, and spatial memory. When the hippocampus is selectively lesioned, humans present with severe spatial memory deficits (Bohbot et al., 1998). It has also been demonstrated that hippocampal lesions in rats produce difficulties in solving spatial navigation tasks (Morris et al., 1982). Visuospatial navigation has been shown to be critically dependent on the hippocampus. Navigating is a cognitively demanding task, and requires individuals to construct a mental representation of the environment with allocentric and egocentric frameworks. The allocentric representation of the environment is dependant on the cognitive map. In other words to be successful at a task one must learn the relations between landmarks (stimulus–stimulus association; Bohbot et al., 2007). According to the cognitive map theory, the main function of the hippocampus is to construct and maintain spatial maps (learning the relationship between environmental landmarks) of the environment (O’Keefe and Nadel, 1978; Kumaran and Maguire, 2005). Therefore, the cognitive map allows a target to be reached in a direct path from any given direction. In contrast, individuals can use stimulus-response learning (Packard et al., 1989) for example using a single landmark as a reference (e.g., at the coffee shop turn left) or make decisions based on their body movement, independent of landmarks in their environment (Iaria et al., 2003). Patients with lesions at the level of the hippocampus are unable to complete spatial allocentric tasks (Bohbot et al., 1998). Recently, it was found in a behavioral study that individuals with schizophrenia exhibit impairments in allocentric memory while egocentric memory remained intact (Weniger and Irle, 2008). Similarly, patients with brain damage to the medial temporal lobe, which includes the hippocampus, are impaired when they spontaneously use an allocentric spatial memory strategy in a dual-solution task (Bohbot et al., 1998). However, similar patients who spontaneously use the stimulus-response strategy are not impaired (Bohbot et al., 2004). Neuroimaging studies have greatly contributed to the literature by providing confirmatory evidence that the hippocampus, together with the parahippocampal gyrus, posterior parietal cortices, medial prefrontal cortex, and striatum are engaged in visuospatial navigation (Aguirre et al., 1996; Maguire et al., 1998; Burgess et al., 2001).

In a previous fMRI study that included the same research participants (schizophrenia and control groups) as those included in the current study, Ledoux et al. (2013) demonstrated an episodic memory deficit in the schizophrenia group. Further, controls performed significantly better on a virtual visuospatial navigation task called the wayfinding task and had significantly increased fMRI activity in the hippocampus compared to these patients while performing the wayfinding task. The wayfinding task used by Ledoux et al. (2013) and in the current study is identical to the one used by Etchamendy and Bohbot (2007), and is modeled after the task used by Hartley et al. (2003). In this task, participants are required to use allocentric representations in order to be successful at finding their way in the environment taking the shortest path. Therefore, in the current paper, we asked whether the wayfinding deficit found in patients with schizophrenia was associated with gray matter (GM) loss in the hippocampus.

Though it is known that the hippocampus is involved in a more general class of memory, such as episodic memory including memory across temporal delays such as trace conditioning (Gruart et al., 2006), explicit memory (Eichenbaum et al., 2012), or relational memory (Clarke et al., 2010), the current paper focuses on the role of the hippocampus in spatial memory (Burgess et al., 2002). The current study used voxel-based morphometry (VBM) to investigate hippocampal GM in patients with schizophrenia and healthy controls previously studied by Ledoux et al. (2013) and examined the relationship between the region of interest in the hippocampus and behavioral performance on the wayfinding task. In this study, we sought to investigate whether performance on the wayfinding task has a predictive relation with the morphological differences in the hippocampus of patients and control participants. Further, we investigated whether the different regions known to be anatomically connected to the hippocampus in a healthy population were associated with the hippocampal GM in the schizophrenia group. It was hypothesized that the patient group would have hippocampal GM differences compared to the control group and that the GM in the hippocampus would play a significant role in performance on the hippocampus-dependent spatial memory task in patients with schizophrenia and in healthy control participants. Finally, in previous studies it was demonstrated that individuals with more GM in the hippocampus also had more GM in an associated network of neuroanatomically connected regions, which include the orbital prefrontal cortex, the parahippocampal cortex, entorhinal and perirhinal cortices, and amygdala (Bohbot et al., 2007; Konishi and Bohbot, 2013). Taking these previous studies into consideration and the disconnectivity theories suggesting connectivity impairments between the hippocampus and prefrontal cortex (Weinberger et al., 1992), it was hypothesized that the patient group would demonstrate differences in the associated network of neuroanatomically connected regions, notably in the orbital prefrontal cortex.

## Materials and Methods

### Participants

A total of 43 study participants who comprised two groups: (1) 21 schizophrenia patients and (2) 22 control participants were recruited for this study. Participants were male and female between 18 and 40 years old inclusively and right-handed (determined by the Handedness Inventory; Oldfield, 1971) due to documented differences in hippocampal areas linked to navigation skills associated with the non-dominant hemisphere. Patients with a primary diagnosis of schizophrenia were recruited from the Outpatient Schizophrenia Clinic at the Royal Ottawa Mental Health Centre. Controls were recruited via newspaper and poster advertisement. The control group was closely matched to the schizophrenia patients in terms of age, sex, and education level. Ethics for the current study were accepted by the Research Ethics Board of the University of Ottawa, Institute of Mental Health Research in Ottawa. Prior to starting the study, all participants signed an informed consent form authorizing the researcher to conduct research with their information. Participants were paid a sum of $75 to take part in the study.

#### Inclusion criteria for the patient group

Participants of this group needed to be clinically diagnosed with schizophrenia by a psychiatrist, therefore they had to meet the Diagnostic and Statistical Manual of Mental Disorders – Fourth Edition criteria (DSM-IV-TR; American Psychiatric Association, 2000). Patients meeting the DSM-IV criteria for schizophrenia, disorganized, undifferentiated, or paranoid subtypes, were eligible to participate in the study; catatonia subtypes and schizoaffective patients were excluded from the study. Patients needed to be clinically stabilized. Stabilization is defined as having had no significant change in symptom severity (i.e., level of severity), and no changes in medication type or dosage or therapeutic methods following a 3-month retrospective chart review determined by their psychiatrist.

#### Exclusion criteria for patient group

Participants with an acute psychotic episode on the total Positive Negative Symptoms Scale (PANSS; Kay et al., 1987) or having a score of 4 on two or more of the following PANSS items (conceptual disorganization, P2; hallucinatory behavior, P3; suspiciousness, P6; unusual thought content, G9) were excluded. Further, participants exhibiting comorbid depressive symptoms [Calgary Depression Scale (CDS) score ≥7; Addington et al., 1990], taking typical antipsychotics, benzodiazepines, or receiving electroconvulsive therapy (ECT) were excluded from this study. Finally, the presence of extrapyramidal symptoms (EPS), or overt signs of tremor or movement disorder could affect the quality of MRI image acquisition. Therefore, patients exhibiting those symptoms were also excluded from this study.

#### Exclusion criteria for control group

Presentation of a psychiatric condition corresponding to an Axis 1 DSM-IV TR diagnosis (using SCID-NP interview) was considered an exclusion criteria. Also, participants reporting a psychiatric history concerning their siblings or other first-degree relatives did not qualify for the study. Finally, having depressive symptoms [Hamilton Scale for Depression (HAMD) score ≥10; Hamilton, 1960] excluded participants from the study.

#### Exclusion criteria common for patient and control groups

Current diagnosis of alcohol abuse or other kinds of dependence in the previous 12 months [Alcohol Use Disorders Identification Test (AUDIT) score ≥8 in men or ≥7 in women; Saunders et al., 1993] or current diagnosis or history of drug abuse or dependence in the past 12 months [Drug and Abuse Screening Test (DAST) score ≥6; Skinner, 1982] were exclusion criterion. Participants with neurological disease, history of head injury, cardiovascular disease, or stroke (determined by Medical Questionnaire) were also excluded. The presence of any non-removable magnetic metal on or in the body (such as cardiac pacemakers, metal prostheses), as determined by the Medical Questionnaire, was also reason for exclusion from the study.

### Material and procedure

#### Clinical assessment

The clinical assessment was a 2 h session separated into two parts. The first hour of the assessment was the clinical interview where the SCID, CDS, or HAMD, PANSS (for patients only) were administered. The second part of the assessment consisted of self-report questionnaires answered by participants. Participants were required to assess themselves on the following questionnaires: AUDIT, DAST.

#### MRI session

The MRI portion of this project occurred in two phases performed on the same day: the pre-scan training phase followed within 1 h by the scan phase. Since familiarity with first-person videogames may have an impact in the virtual reality task performance, before the pre-scan training participants were asked about their video game habits [e.g., what type(s) of video game played]. During the pre-scan training, participants were first familiarized with the keyboard to ensure their ability to maneuver through the environment. This was done in a virtual environment different from the virtual town used for the experiment. They then navigated in the virtual town created with the game editor of a commercially available computer game (Unreal Tournament 2003; Epic Games, Raleigh, NC, USA). The virtual town is a visually complex computer-based environment, which includes several roads, intersections, and buildings, in addition to distinct landmarks (easily recognizable, labeled locations such as a school or a hospital). Participants engaged in a free exploration of the town for 20–30 min. During exploration, participants were required to encounter every landmark twice and to travel along all roads. The path taken and the amount of time participants visited each landmark was recorded. The free exploration provided an opportunity for participants to encode and construct a cognitive map of the environment by building relationships between landmarks in the town. Participants were not permitted sufficient exploration time to form habitual routes between landmarks. Creating the paradigm using a modified video game framework provided participants with a first-person perspective while navigating. Following the training outside the MRI, participants were scanned while performing tasks based on the navigation paradigm. In the MRI, participants underwent a 15 min T1 structural scan. During fMRI scanning which followed, participants were required to complete the Navigation Task. For each eight navigation trials, participants were placed in one location in the city (e.g., school) and were required to navigate from there to another landmark (e.g., movie theater) within the city. Successful completion of this task required taking the shortest route by deriving it from a cognitive map, a task that critically requires the hippocampus. During completion of the navigation trials, participants were timed and their precise paths were recorded on a 2D aerial view of the town.

Magnetic resonance imaging scans were acquired using a 1.5 Tesla Siemens Magnetom Symphony. The protocol generated T1-weighted image volumes with a 1 mm isotropic resolution using a three-dimensional spoiled gradient echo acquisition with sagittal volume excitation (repetition time, 22; echo time, 9.2; flip angle, 30°; field of view, 256 mm; 160 1 mm sagittal slices). An MRI-compatible virtual reality system, Silent Vision^TM^ Model SV-7021 Fiber Optic Visual System with In Control Software (Avotec, Inc.), was acquired for this study, as well as a four button fiber optic touch pad.

### Data analyses

Participants were matched according to their age, sex, and level of education for all analyses in this study. Behavioral data (demographics and navigation performances) were analyzed using SPSS (SPSS, 2008) software. Neuroimaging data was analyzed with Statistical Parametric Mapping software (SPM8, 2008) and the VBM8 toolbox (http://dbm.neuro.uni-jena.de/vbm/).

#### Behavioral data

The first hypothesis of a significant difference between patient and control groups within the behavioral navigation performance was analyzed with a multivariate ANOVA. Variables considered for the analysis of navigation performance were Accuracy (i.e., percentage of target locations reached), Percent error (i.e., percentage of extra distance traveled + distance remaining to reach the goal compared to shortest distance needed to reach goal location), and mean time.

$$\mathrm{Percent error :}\;\left(\left(x+z\right)-y∕\left(x+z\right)\times \mathrm{100}\right)$$

(i.e., *x* = total distance traveled, *z* = distance remaining to reach the goal, *y* = shortest distance to goal). The *z* variable was included to account for incomplete trials where the target landmark was not reached. Since incomplete trials by definition are missing part of the way to the goal location, the *z* variable was made to include this missing distance. Therefore, the shortest distance from the end point at which the trial was interrupted to the goal location is added to the distance traveled (*x* + *z*).

#### Voxel-based morphometry and statistical analysis

We applied VBM as implemented in the VBM8 toolbox with default parameters. Images were bias-corrected, tissue classified, and registered using linear (12-parameter affine) and non-linear transformations (warping), within a unified model (Ashburner and Friston, 2005). Subsequently, analyses were performed on GM and white matter (WM) segments, which were multiplied by the non-linear components derived from the normalization matrix in order to preserve actual GM and WM values locally (modulated GM and WM average). Finally, the modulated volumes were smoothed with a Gaussian kernel of 12 mm full width at half maximum (FWHM). GM, WM, and Cerebral Spinal Fluid (CSF) maps were combined for total intracranial volume (TIV). Voxel-wise GM and WM differences between schizophrenia patients and controls were examined using independent-sample *t*-tests. In order to avoid possible edge effects between different tissue types, we excluded all voxels with GM or WM values of less than 0.1 (absolute threshold masking).

A first regression was performed on GM and navigation performance to determine whether GM regressed with the percentage errors made during the task, accuracy, and time.

The purpose of our study was primarily focused on the hippocampal structure, and secondly the prefrontal cortex and caudate nucleus. Region of interests (ROI) analyses were restricted to the hippocampus, parahippocampal gyrus, caudate nucleus, superior medial, and orbital prefrontal cortices. We created a mask for all specified ROI with the Pick Atlas extension (Maldjian et al., 2003) using the automated anatomical labeling atlas (Tzourio-Mazoyer et al., 2002). This mask was then inserted as the explicit mask in the VBM factorial analyses. Within the masks, significance was set at a threshold of uncorrected *p* < 0.001, with a cluster-wise correction at *p*FWE = 0.05 and a set cluster size larger than 10 voxels.

Pearson correlations were performed between subtracted hippocampal GM regions at the peak voxel (MNI space *x* = 24, *y* = −21, *z* = −15 and *x* = −24, *y* = −21, *z* = −18) and the navigation behavioral variables in both groups.

Results of the first VBM regression were used to regress the GM value at the peak voxel (MNI space coordinates, *x* = 24, *y* = −21, *z* = −15) in the hippocampus against the entire MRI volume in the control and patient groups. This second regression tested and compared whether GM in the network of regions known to be anatomically linked to the hippocampus covaried with hippocampal GM in patients. A similar regression was also performed in Bohbot et al. (2007) showing that navigational strategies correlate with GM in the hippocampus or caudate of healthy participants. In turn, GM in the hippocampus correlated with a network of brain regions, known to be anatomically connected (i.e., orbital prefrontal cortex, the parahippocampal, entorhinal and perirhinal cortices, and amygdala).

## Results

### Demographics

Twenty-one patients with schizophrenia and 22 healthy controls qualified and completed the study. All together, 20 pairs were matched on age, sex and education. However, one patient (male age 30) and three controls (one female age 22 and two males age 19 and 37) were not matched because their respective matched partner was withdrawn from the study due to miscellaneous difficulties (e.g., WM abnormalities, experiencing excessive anxiety in the scanner). Therefore, matching of our samples was done at a group level. Participant demographics are demonstrated in Table 1. No significant differences were found in the main matching criteria variables: age, sex, education, and no significant differences were found in IQ scores, experience with first-person videogames, or mean number of time participants visited the landmarks during the learning phase (visited landmarks), *p* > 0.05 (Table 1). Patients’ mean PANSS global score of 64 represents a very moderate severity and slightly predominant but not marked negative symptoms (18.4 vs. 15.4), in summary, a moderate and stabilized population.

**Table 1:** **Participant demographics**.

	Controls (*n* = 22; SD)	Patients (*n* = 21; SD)
Sex (F/M)	6/16	5/16
Age (years)	30.45 (1.25)	32.05 (1.08)
Education (years)	16.68 (2.64)	15.10 (2.09)
IQ (NART)	111.32 (1.67)	109.09 (1.47)
Played first-person videogame (yes/no)	13/9	13/8
Visited landmarks	20.64 (4.76)	21.38 (5.36)
Age of onset (years)		20.52 (4.81)
Duration of illness (years)		11.38 (4.93)
PANSS total		64 (13.0)
PANSS positive		15.14 (4.33)
PANSS negative		18.14 (5.68)
PANSS general score		30.71 (6.93)

**p* > 0.05 on all measures*.

### Behavioral navigation scores

**Figure 1:**
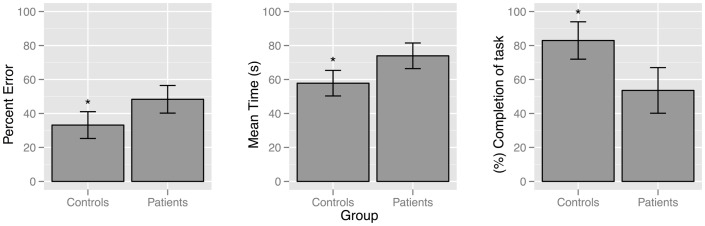
**Bar plot demonstrating differences between patient and control groups for the behavioral navigation variables: percent error [*F* (1,41) = 9.64, *p* = 0.003]; mean time [*F* (1,41) = 12.28, *p* = 0.001]; and accuracy [*F* (1,41) = 15.47, *p* = 0.000]**.

Compared to controls, patients with schizophrenia found fewer target landmarks [*F*(1, 41) = 15.47, *p* = 0.000], traveled longer distances [*F*(1, 41) = 9.639, *p* = 0.003], and took more time [*F*(1, 41) = 12.28, *p* = 0.001] to find target landmarks, Figure 1.

### Regional GM reduction in patients compared to control participants

**Figure 2:**
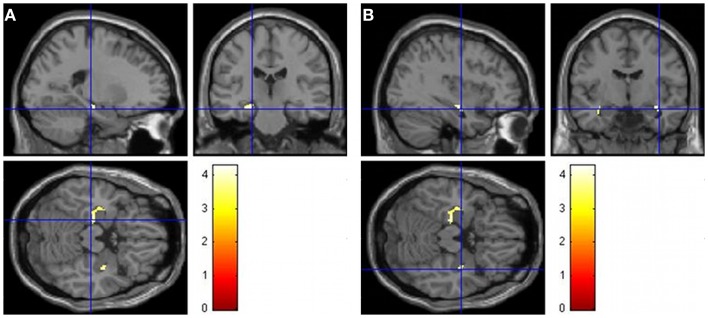
**Images demonstrating GM differences of the hippocampus when contrasting controls and patients (controls > patients)**. The *t* maps are superimposed onto an anatomical brain and displayed in the sagittal, coronal, and horizontal planes. **(A)** GM differences in the left hippocampus when contrasting the control group to the patient group (−22, −22, −17). **(B)** GM differences in the right hippocampus when contrasting the control group to the patient group right (36, −9, −15).

Groups did not differ in overall TIV (*t*_41_ = −0.96, *p* = 0.34). In order to investigate whether there were whole brain GM and WM differences, independent-sample *t*-tests were performed. Since there was no significant difference in age or TIV, these variables were not used as covariables. Significant differences in GM (Table 2 and Figure 2) were found between groups (Controls > Patients). WM analysis showed significant differences between controls and patients in the right middle frontal cortex (MNI coordinates: 33, 18, 21; *t* = 4.48) and right frontal inferior triangularis gyrus (MNI coordinates: 30, 39, 21; *t* = 4.02), as well as in the ROI of the posterior left hippocampus (−12, −33, 9; *t* = 4.22).

**Table 2:** **Reductions of GM volume in patients with schizophrenia compared to healthy controls (controls > patients)**.

Region	Side	MNI coordinates			*Z* value	*t* value
		*x*	*y*	*z*	
Insula	L	−28	20	−2	5.98	7.61
Insula	R	34	15	−20	4.60	5.29
Middle frontal gyrus	R	34	36	18	4.50	5.15
Gyrus rectus	L	−2	39	−18	4.49	5.15
Frontal inferior triangular gyrus	R	39	33	3	4.48	5.12
**ROI**						
Hippocampus	L	−22	−22	−17	3.81	4.21
Hippocampus	L	−36	−12	−17	3.39	3.67
Hippocampus	R	38	−9	−15	3.80	4.18
Hippocampus	R	30	−15	−12	3.23	3.48
Caudate	R	22	24	−2	3.90	4.32
Front inferior orbital	L	−30	27	−5	5.29	6.39
Frontal superior orbital	L	−26	14	−14	5.24	6.30
Frontal superior orbital	R	22	18	−14	4.91	5.78
Frontal inferior orbital	R	34	14	−20	4.51	5.16
Frontal medial orbital	L	−3	39	−15	4.39	5.00
Frontal medial orbital	R	3	68	−2	4.20	4.73

*ROI investigation at threshold 0.001 uncorrected, *P*_FWE_ = 0.05 cluster-wise correction*.

#### First regression: association between GM and behavioral variables

**Figure 3:**
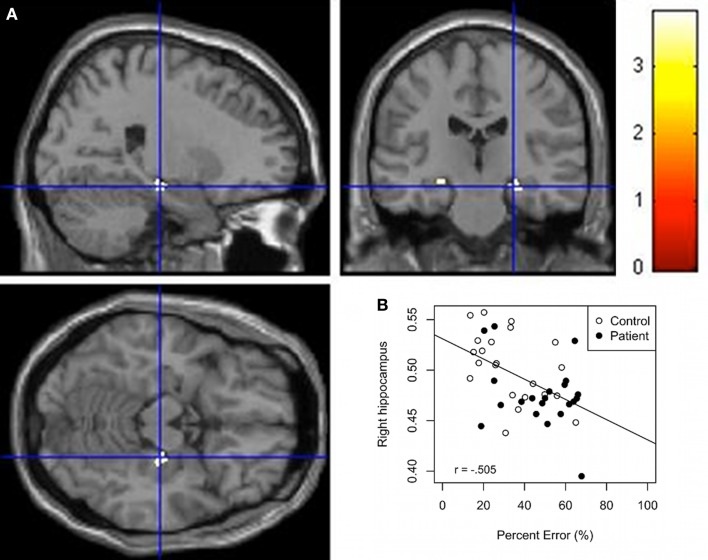
**(A)** VBM Regression analysis of right hippocampus GM against percent error on the wayfinding task (*p*FWEcorr. < 0.05). The *t* maps are superimposed onto an anatomical brain and displayed in the sagittal, coronal, and horizontal planes. **(B)** Scatter plot regression of right hippocampal cluster at seed voxel (24, −21, −15; *p* < 0.001) with Percent error on the wayfinding task. Pearson correlation between right hippocampus GM and percept error for the control group (*r* = −0.484, *p* = 0.01) and patient group (*r* = −0.318, *p* = 0.08).

Whole group regressions were performed with GM and the behavioral variables. Results (Table 3) indicate an inverse association between the behavioral variables (Time and Percent error) and the right hippocampus and right parahippocampal cortex GM. Along the same lines, a positive correlation was found between the right parahippocampal cortex GM and Accuracy. Scatter plots of Percent error against hippocampus GM derived from this analysis shows that the control group forms two separate groups (Figure 3B) even if the scores in each axis seem almost normally distributed. A subsequent analysis confirms a significant difference between both subgroups for the variables percent error and right hippocampus GM (*F* = 40.88, *p* < 0.01; *F* = 24.24, *p* < 0.01). In addition, Figures 3A,B shows that patients have less hippocampal GM and poorer navigation performance. Regression analyses for the separated groups show a significant negative correlation (*p*_uncorr._ < 0.001) between selected brain areas including predominantly the right hippocampus, right parahippocampal cortex, and left caudate GM and Time and Percent error in controls. In addition, we found a significant negative correlation (*p*_uncorr_ = 0.05) between predominantly the left hippocampus and right parahippocampal cortex GM and time and percent error in patients. A positive correlation was found between the right parahippocampal cortex and accuracy in controls and the right parahippocampal cortex and left hippocampus and accuracy for the patient group (*p*_uncorr._ < 0.01). Bivariate Pearson correlations support these results where the right hippocampus seed region correlated significantly with percent error and time in controls (*r* = −0.484, *p* = 0.01; *r* = −0.482, *p* = 0.01) but not in patients (*r* = −0.318, *p* = 0.80; *r* = −0.277, *p* = 0.11) and the left hippocampus seed region correlated significantly with percent error and time in the patient group (*r* = −0.401, *p* = 0.036; *r* = −0.429, *p* = 0.026) but not in the control group (*r* = −0.344, *p* = 0.06; *r* = −0.235, *p* = 0.15).

**Table 3:** **All participants**.

Condition	Region	MNI coordinates			*K*_E_	*t* value	*Z* value
		*x*	*y*	*z*	
Accuracy (+)	R parahippocampal cortex	33	−34	−15	80	3.89	3.57
	R caudate	10	16	−11	80	4.00	3.65
	L caudate	−10	18	−11	104	3.78	3.48
Time (−)	R parahippocampal cortex	33	−34	−15	274	4.62	4.12
	R hippocampus	24	−21	−14	88	3.68	3.40
	R caudate	10	15	−12	74	4.13	3.76
	L caudate	−8	−16	−11	139	3.84	3.53
	L frontal inferior orbital	−34	23	−9	760	4.27	3.86
	L frontal superior orbital	−22	17	−15	760	4.20	3.80
	R frontal superior medial	8	35	60	193	3.80	3.49
Percent error (−)	R parahippocampal cortex	30	−31	−17	222	4.12	3.74
	R hippocampus	24	−21	−15	78	3.82	3.51

*ROI investigation at threshold 0.001 uncorrected, *P*_FWE_ = 0.05 cluster-wise correction and *k* > 10, (R > right, L = Left, (+) = positive, (−) = negative)*.

#### Second regression: regression at the seed voxel of the hippocampus

**Figure 4:**
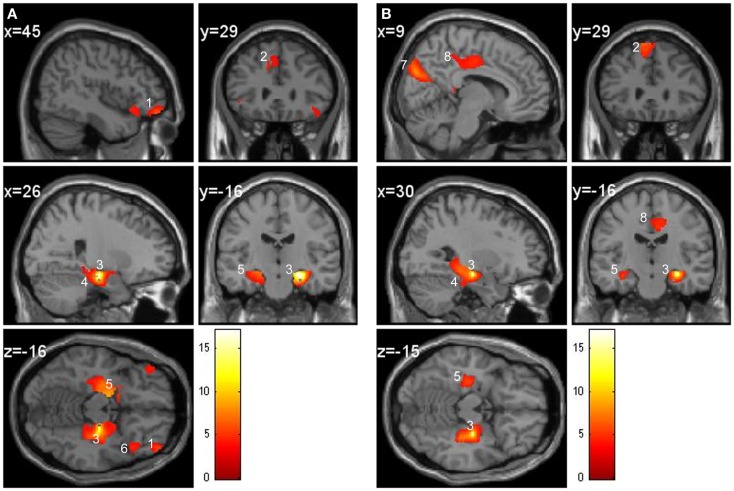
**Regression at the seed voxel of the right hippocampus (24, −21, −15) in controls (A) and in patients (B)**. The *t* maps are superimposed onto an anatomical brain and displayed in the sagittal, coronal, and horizontal planes. **(A)** Right hippocampus (region 3) in controls covaries significantly with the frontal middle orbital cortex (region 1), superior medial gyrus (region 2), right parahippocampal cortex (region 4), and left hippocampus (region 5). Inferior orbital cortex (region 6) does not significantly correlate with the right hippocampus. **(B)** Right hippocampus (region 3) of patients covaries significantly with the cuneus (region 7), middle cingulate gyrus (region 8), superior medial frontal gyrus (region 2), parahippocampal cortex (region 4), and left hippocampus (region 5).

In the control group, the regions covarying significantly with the right hippocampus were the bilateral parahippocampal cortex, left entorhinal cortex, contralateral hippocampus, amygdala, frontal middle orbital cortex, and frontal superior medial gyrus GM (Figure 4A). For the patient group, the bilateral parahippocampal cortex, contralateral hippocampus, amygdala, cuneus, frontal superior medial gyrus, and middle cingulate gyrus GM covaried with the right hippocampus (Figure 4B). To summarize, in the patient group the right hippocampus did not covary with the entorhinal and middle orbital cortex as within the control group but did covary with the cuneus and middle cingulate gyrus, which was not the case in the control group.

## Discussion

The goal of this study was to investigate whether GM in the hippocampus would predict performance on a hippocampus-dependent spatial memory task in patients with schizophrenia and in control participants. Furthermore, this study sought to explore whether regions known to be anatomically connected with the hippocampus covaried with the right hippocampus GM region derived from the VBM regression analysis. To assess spatial memory, a wayfinding task was utilized where participants explored a virtual town and had to remember the location of several landmarks. Spatial memory was tested by asking participants to navigate from one landmark to another by taking the shortest route possible. It was hypothesized that the patient group would have a smaller hippocampal GM average compared to the control group and the size of the hippocampus could predict the performance on the navigation task.

During the navigation task, individuals with schizophrenia reached the target goal less often, took more time, and deviated from the shortest route possible significantly more than controls. When comparing controls to patients, GM volumetric analysis revealed significantly lower GM average in the hippocampus of the patient group. This analysis also revealed GM average differences in the insula, middle, and inferior frontal gyrus, gyrus rectus, caudate nucleus, and frontal orbital cortex.

Whole group regression analysis revealed increased latency and deviation from the shortest route were associated with a decrease in the right hippocampus GM, indicating that more GM in the hippocampus is associated with better performance (finding the landmark by making fewer errors and taking less time to complete the task). The anterior right hippocampus has also been associated with performance in navigation in other studies. Bohbot et al. (2007) found that individuals using spontaneous spatial strategies had greater GM in the hippocampus compared to individuals using non-spatial strategies. As seen in the scatter plot (Figure 3) there appears to be two clusters in the control group, the good performers with greater right hippocampal GM and poor performers with lower right hippocampal GM. In support of these findings, Etchamendy and Bohbot (2007) found that approximately 50% of their participants used a spontaneous spatial strategy and those who maintained that strategy on the 4-on-8 virtual maze (the task used to dissociate spatial and response strategies) performed significantly better on the wayfinding task than participants who used a response strategy navigating from their starting position. Spatial learners on the 4-on-8 virtual maze also had significantly more GM in the hippocampus than response learners, which would be consistent with the current results. Head and Isom (2010) also demonstrated more GM in individuals who were better at the wayfinding task. Clearly, the current results show an association between hippocampus GM and the ability to learn the relations between the environmental landmarks in order to perform the task successfully.

Compared to controls participants, patients performed significantly worse at the navigation task and had a smaller hippocampus. Low GM volumes have been previously reported in schizophrenia (Wright et al., 2000) and also in first episode groups (Pantelis et al., 2003). Lower GM volumes may be a risk factor for schizophrenia. These anatomical hippocampal anomalies may be the cause of spatial learning impairments and other important cognitive deficits seen in schizophrenia, such as an episodic memory deficit. We previously demonstrated significant differences between both groups, whereby patients made more errors at the immediate and delayed recall of the family picture subtest of the Weschler memory test (Wechsler, 1987) compared to the control group (Ledoux et al., 2013), which is, an assessment that specifically tests the ability to associate together the context and content of an event. The literature postulates that the episodic memory deficit seen in schizophrenia might be mediated by a contextual binding deficit (Boyer et al., 2007), the ability to make associations between the content (“what”) and the contextual features (the “where” and “when”) of an event. Since the hippocampus is critical for contextual binding in episodic memory (Burgess et al., 2002; Maguire and Frith, 2004), it seems appropriate to use the wayfinding task which assesses similar mechanisms [i.e., in order to reach the target location, participants are required to learn the relations between the environmental landmarks (association of the event with its spatial context)].

Interestingly, the wayfinding-hippocampus GM relation in the patient group (Figure 3) shows a cluster, which overlaps with the poor performers in the control group, i.e., with the lower right hippocampus GM group. This indicates that both the patient and the poor performer control groups are in the lower range in terms of GM in the hippocampus and performance on the spatial navigation task. Since, patients in their prodromal and first psychotic episode groups have low hippocampus GM (Pantelis et al., 2003), data in the literature suggest that healthy participants with low hippocampus GM, similar to those in our poor performer control group, may be at risk for neurological and psychiatric disorders such as schizophrenia. The current results suggest that the wayfinding task may be sensitive to abnormalities in the hippocampus for different types of populations.

The prefrontal cortex, striatum, and parahippocampal gyrus are all regions that have been implicated in previous visuospatial navigation studies (Aguirre et al., 1996; Bohbot et al., 1998; Maguire et al., 1998; Burgess et al., 2002; Hartley et al., 2003; Iaria et al., 2003). In this study, it was found that the GM of these regions was also associated with the performance on the wayfinding task.

Regression between the seed region in the right hippocampus and the entire brain demonstrates that the parahippocampal cortex, entorhinal cortex, contralateral hippocampus, amygdala, frontal middle orbital cortex, and frontal superior medial gyrus GM regions correlate with the hippocampus in the control group. In other words, when GM is greater in the hippocampus it will also be greater in the above mentioned regions. These regions are known to be anatomically connected to the hippocampus and were also found to positively covary with the hippocampus in similar navigation studies (Bohbot et al., 2007; Konishi and Bohbot, 2013). Contrary to the control group, in the patient group the cuneus and cingulate gyrus also covaried with the right hippocampus but not the entorhinal and frontal orbital cortices. These regions might be recruited to compensate for the structural and functional deficit seen in schizophrenia while navigating. As previously mentioned the frontal orbital and hippocampal regions were found to have less GM in the patient group. Empirical evidence supports a connection involving neuroanatomical projections from the CA1 and subiculum fields to the medial prefrontal and orbital frontal cortices (Thierry et al., 2000). The hippocampo-prefrontal pathway represents one of the major factors in learning and memory (Laroche et al., 2000). These results provide support to the hippocampal-prefrontal connectivity hypothesis in schizophrenia, which suggests that the prefrontal deficit (such as executive functioning) may in fact be more closely linked to a temporal lobe deficit or associated with connectivity deficits between the temporal lobe and the prefrontal cortex (Weinberger et al., 1992).

Maguire et al. (2000), found that taxi cab drivers, whom have extensive navigation experience, had greater hippocampal volumes than the control group, and a recent study by Lerch et al. (2011), demonstrated in mice that spatial memory training causes neuroanatomical volume changes in the hippocampus. The wayfinding task employed in this study seems to be particularly sensitive to the hippocampus. Training individuals with schizophrenia on hippocampal-dependent tasks, could potentially be used as a form of therapy to help improve the function and structure of the hippocampus, potentially alleviating cognitive deficits seen in this population such as episodic memory problems and executive dysfunction.

Finally, for future research, a study with a larger sample of participants in each group would be a better sample to generalize these results to this population (Steen et al., 2007).

## Conclusion

In the current study, we investigated whether performance in a wayfinding task could predictably be related to GM in a healthy control group and in a schizophrenia patient group and explore whether the same GM regions in both groups covaried with the hippocampus. Control participants successfully found more target locations in the environment than patients, took less time to complete the task, and made fewer errors compared to patients. Controls had a greater hippocampal GM average than patients. Whole group performance was significantly related to the right hippocampus. Patients’ poor performance, contextual binding deficit, and reduced hippocampal activity while performing the wayfinding task, as demonstrated in our previous study, may be attributed to hippocampal anomalies. However, a greater sample size would be necessary to confirm these results. The second VBM regression analysis demonstrated that orbital frontal cortex does not relate with the hippocampal GM in the patient group, a result congruent with the hippocampal-prefrontal connectivity hypothesis of schizophrenia. Results of this study demonstrate that individuals with schizophrenia have a hippocampal disorder and directly targeting the hippocampal structure might be important for improving cognitive impairments.

## Authors Contribution

Andrée-Anne Ledoux contributed substantially to the conception and design of the experiment design and manuscript; she did the acquisition/collection of the clinical, cognitive, and neuroimaging data, helped in the creation of the neuroimaging paradigm (virtual town), analyzed and interpreted the data, and wrote the paper. Patrice Boyer contributed substantially to the conception or experimental design and hypotheses; he also trained authors on the acquisition of clinical data, revised the work critically for important intellectual content, approved final version of the document, and ensured accuracy and integrity of clinical, navigation, and neuroimaging component of the work and that they are appropriately investigated and resolved. Jennifer L. Phillips contributed at the conception of the experimental design, recruited participants, collected clinical, and neuroimaging data, edited, revised and approved the final version of the manuscript. Andra Smith contributed at the conception of the experimental neuroimaging design, ensured accuracy, and integrity in the neuroimaging data and approved the final version of the paper. Alain Labelle recruited schizophrenia patients, revised the work critically for important intellectual content and approved the final version of the paper. Véronique D. Bohbot contributed substantially to the conception or design of the work and hypotheses; helped with the acquisition parameters of the neuroimaging data, trained the first author on the creation of the neuroimaging paradigm, revised the work critically for important intellectual content, approved the final version to be published and ensured accuracy and integrity of navigation and neuroimaging work and made sure the work was appropriately investigated.

## Conflict of Interest Statement

The authors declare that the research was conducted in the absence of any commercial or financial relationships that could be construed as a potential conflict of interest.
